# In situ expression of (*R*)-carbonyl reductase rebalancing an asymmetric pathway improves stereoconversion efficiency of racemic mixture to (*S*)-phenyl-1,2-ethanediol in *Candida parapsilosis* CCTCC M203011

**DOI:** 10.1186/s12934-016-0539-y

**Published:** 2016-08-17

**Authors:** Rongzhen Zhang, Lei Wang, Yan Xu, Hongbo Liang, Xiaotian Zhou, Jiawei Jiang, Yaohui Li, Rong Xiao, Ye Ni

**Affiliations:** 1 Key Laboratory of Industrial Biotechnology of Ministry of Education and School of Biotechnology, Jiangnan University, Wuxi, 214122 People’s Republic of China; 2 National Key Laboratory for Food Science, Jiangnan University, Wuxi, 214122 People’s Republic of China; 3 Center for Advanced Biotechnology and Medicine, Rutgers University, Piscataway, NJ 08854 USA; 4 School of Biotechnology, Jiangnan University, 1800 Lihu Avenue, Wuxi, 214122 People’s Republic of China

**Keywords:** (*R*)-carbonyl reductase, *Candida parapsilosis*, In situ expression, Racemic 1-phenyl-1,2-ethanediol, Stereoconversion, (*S*)-1-phenyl-1,2-ethanediol

## Abstract

**Background:**

*Candida parapsilosis* (*R*)-carbonyl reductase (RCR) and (*S*)-carbonyl reductase (SCR) are involved in the stereoconversion of racemic (*R,S*)-1-phenyl-1,2-ethanediol (PED) to its (*S*)-isomer. RCR catalyzes (*R*)-PED to 2-hydroxyacetophenone (2-HAP), and SCR catalyzes 2-HAP to (*S*)-PED. However, the stereoconversion efficiency of racemic mixture to (*S*)-PED is not high because of an activity imbalance between RCR and SCR, with RCR performing at a lower rate than SCR. To realize the efficient preparation of racemic mixture to (*S*)-PED, an in situ expression of RCR and a two-stage control strategy were introduced to rebalance the RCR- and SCR-mediated pathways.

**Results:**

An in situ expression plasmid pCP was designed and RCR was successfully expressed in *C. parapsilosis*. With respect to wild-type, recombinant *C. parapsilosis*/pCP-RCR exhibited over four-fold higher activity for catalyzing racemic (*R,S*)-PED to 2-HAP, while maintained the activity for catalyzing 2-HAP to (*S*)-PED. The ratio of *k*_*cat*_/*K*_*M*_ for SCR catalyzing (*R*)-PED and RCR catalyzing 2-HAP was about 1.0, showing the good balance between the functions of SCR and RCR. Based on pH and temperature preferences of RCR and SCR, a two-stage control strategy was devised, where pH and temperature were initially set at 5.0 and 30 °C for RCR rapidly catalyzing racemic PED to 2-HAP, and then adjusted to 4.5 and 35 °C for SCR transforming 2-HAP to (*S*)-PED. Using these strategies, the recombinant *C. parapsilosis*/pCP-RCR catalyzed racemic PED to its (*S*)-isomer with an optical purity of 98.8 % and a yield of 48.4 %. Most notably, the biotransformation duration was reduced from 48 to 13 h.

**Conclusions:**

We established an in situ expression system for RCR in *C. parapsilosis* to rebalance the functions between RCR and SCR. Then we designed a two-stage control strategy based on pH and temperature preferences of RCR and SCR, better rebalancing RCR and SCR-mediated chiral biosynthesis pathways. This work demonstrates a method to improve chiral biosyntheses via in situ expression of rate-limiting enzyme and a multi-stage control strategy to rebalance asymmetric pathways.

**Electronic supplementary material:**

The online version of this article (doi:10.1186/s12934-016-0539-y) contains supplementary material, which is available to authorized users.

## Background

**Fig. 1:**
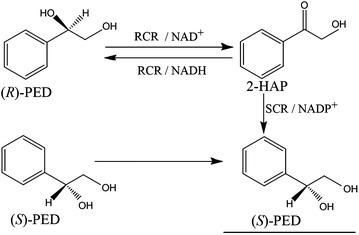
The stereoinversion of, (*R,S*)-PED to its (*S*)-isomer by RCR and SCR from *C. parapsilosis*. An NAD^+^-dependent RCR catalyzes the reversible reaction between (*R*)-PED and 2-HAP. An NADPH-linked SCR transforms the intermediate 2-HAP to (*S*)-PED. The oxidation of (*R*)-PED to 2-HAP is rate-limiting step during the stereoinversion of racemic (*R,S*)-PED to its (*S*)-isomer. The desired product (*S*)-PED is *underlined*. PED, 1-phenyl-1,2-ethanediol; RCR, (*R*)-carbonyl reductase; SCR, (*S*)-carbonyl reductase

Optically active alcohols are versatile chiral building blocks for organic synthesis in a variety of different industries. Considerable research efforts have recently focused on the preparation of chiral compounds using enzyme-catalyzed bioprocesses [1, 2]. Stereospecific carbonyl reductases are good candidates to catalyze prochiral ketones to the corresponding enantiopure alcohols [3–5]. For example, *Candidaparapsilosis* CCTCC M203011 contains (*R*)- and (*S*)-carbonyl reductases (RCR and SCR), which catalyze the biotransformation of the valuable, optically active (*S*)-1-phenyl-1,2-ethanediol (PED) (Fig. 1), which can be used for synthesis of an antidepressant drug, fluoxetine, which extends the effects of 5-hydroxy tryptamine through selective inhibition for the extraction of 5-hydroxy tryptamine in central nervous system [6, 7]. The enzymatic biosynthesis of (*S*)-PED involves two sequential redox reactions: (i) RCR catalyzes reversible conversion of 2-hydroxyacetophenone (2-HAP) to (*R*)-PED; and (ii) SCR catalyzes reduction of the intermediate 2-HAP to (*S*)-PED. The asymmetric flux of this process is controlled by the activity of the RCR and SCR enzymes, with the initial RCR-catalyzed reaction being the rate-limiting step. It was reported that wild-type (WT) *C*. *parapsilosis* and recombinant *Escherichia coli* containing RCR and SCR biotransform (*R*)-PED to the corresponding (*S*)-isomer with low yields, most likely because of the unbalanced efficiency of turnover of the enzymes [8, 9].

Many carbonyl reductases have been engineered in different host cells to improve their catalytic activity and/or protein production [10, 11]. However, some recombinant carbonyl reductases have been reported to exhibit low or no enzymatic activity, which could be caused by incorrect protein folding or lack of post-translational modifications [11, 12]. For example, the specific activities of carbonyl reductase and pyranose dehydrogenase expressed in yeast were much higher than those in *E. coli* [13, 14].

In our previous study, we successfully expressed the RCR and SCR enzymes of *C. parapsilosis* in different host cells, including *E*. *coli* [15], *Saccharomyces cerevisiae* [16] and *Pichia pastoris* [13, 17]. Unfortunately, these recombinant enzymes produced the desired chiral PED with low yields over a long reaction time of 48 h [13, 15–17]. The recently developed in situ expression technique offers a new approach for increasing protein activity and production since the natural host supplies the better environment for protein-folding than heterogenous host. Yang et al. efficiently produced 2,3-butanediol using an in situ expression system containing glyceraldehyde-3-phosphate dehydrogenase and 2,3-butanediol dehydrogenase in *Bacillus amyloliquefaciens* [18]. Piskin et al. proved that in situ expression of IL-23 by keratinocytes in healthy skin and psoriasis lesions enhanced expression in psoriatic skin [19]. Kasai et al. reported increased expression of type I 17β-hydroxysteroid dehydrogenase enhances in situ production of estradiol in uterine leiomyoma [20]. The in situ expression of RCR in *C. parapsilosis* may provide the appropriate environment for correct protein-folding and improve its catalytic activity. *C. parapsilosis* is the major fungal pathogen and is of significant medical and biotechnological importance, so the successful genetic manipulation of this fungus will ultimately lead to the identification of valuable new biotechnological processes [21]. The genetic manipulation of *Candida* species has been challenging because they lack natural plasmids [22, 23]. The reported gene disruption method in *Candida* strains by Reuß et al. [24–26] and the molecular genetics of *Candidaalbicans* by De Backer et al. [27] might supply convenient technical information for in situ protein expression in *Candida* species.

In this work, to improve the biotransformation efficiency of racemic (*R,S*)-PED to its (*S*)-isomer by *C. parapsilosis* through improving RCR activity to rebalance the functions of RCR and SCR, we designed a special vector pCP for in situ expression of RCR in *C. parapsilosis.* The catalytic efficiency of RCR was significantly enhanced, better rebalancing the RCR and SCR functions. Based on pH and temperature preferences of RCR and SCR, we proposed a two-stage control strategy to rebalance RCR and SCR-mediated asymmetric biosynthetic pathway. The in situ expression system *C. parapsilosis*/pCP-RCR catalyzed racemic PED to (*S*)-PED with high optical purity of 98.8 % and a high yield of 48.4 %. More importantly, the biotransformation process was decreased from 48 to 13 h. This work provides a method for improving chiral biosynthesis efficiency through the in situ expression of a rate-limiting enzyme and a two-stage pH and temperature control strategy to rebalance multi-enzyme mediated asymmetric pathways.

## Results

### Design of in situ expression system of RCR in *C. parapsilosis*

**Fig. 2:**
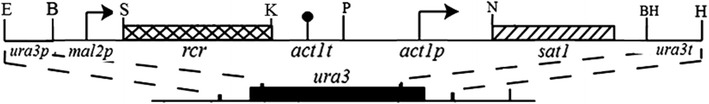
Construction of an expression cassette for RCR in *C. parapsilosis*. *ura3p* (upstream sequence of *ura3* gene) and *ura3t* (downstream sequence of *ura3* gene) fragments were amplified using primers URA3p_1/URA3p_2 and URA3t_1/URA3t_2, and used as homologous regions for insertion into the genome. *mal2p* (upstream sequence of *mal2* gene) and *act1p* (upstream sequence of *act1* gene) fragments were amplified using primers MAL2p_1/MAL2p_2 and ACT1p_1/ACT1p_2, and used as the promoters for expression of the *rcr* and *sat1* genes, respectively. *act1t* (downstream sequence of *act1* gene) and *ura3t* (downstream sequence of *ura3* gene) fragments were amplified using primers ACT1t_1/ACT1t_2 and URA3t_1/URA3t_2, and used as the terminators for the *rcr* and *sat1* genes, respectively. Restriction sites are unique. *E Eco*RI, *B* *Bgl*II, *S* *Sac*I, *K* *Kpn*I, *P* *Pst*I, *N* *Not*I, *BH* *Bam*HI, *H* *Hin*d III

The in situ expression plasmid pCP-*rcr* containing 6× His-tagged RCR was constructed using standard techniques described in the “Methods” section. Briefly, a 5′-terminal 548-bp fragment of a *mal*2 ortholog was used to drive *rcr* expression, and a 3′-terminal 388-bp fragment of an *act*1 ortholog to terminate *rcr* expression. An *act1* promoter (1141 bp) and an *ura*3 terminator (508 bp) were used to control *sat*1 expression, which conferred nourseothricin resistance in *C*. *parapsilosis* [24, 28]. Since seven DNA fragments (*ura3p*, *mal2p*, *rcr*, *act1t*, *act1p*, *sat1* and *ura3t*) were cloned into pUC57 simultaneously to construct the recombinant pCP-*rcr* (Fig. 2), some of their restriction enzyme recognition sites were mutated, and the enzyme digestion and overlap-extension PCR techniques were both used. The length of expression cassette *ura3p*-*mal2p*-*rcr*-*act1t*-*act1p*-*salt*-*ura3t* was about 4.5 kb. Recombinant plasmid pCP-*rcr* was transformed into *E. coli* DH5*α* competent cells to construct *E. coli* DH5*α*/pCP-*rcr*. Subsequent nucleotide sequencing confirmed that the expression cassette *ura3p*-*mal2p*-*rcr*-*act1t*-*act1p*-*salt*-*ura3t* had been correctly cloned into pCP-*rcr* plasmid.

### In situ expression of RCR rebalanced the RCR and SCR functions

**Fig. 3:**
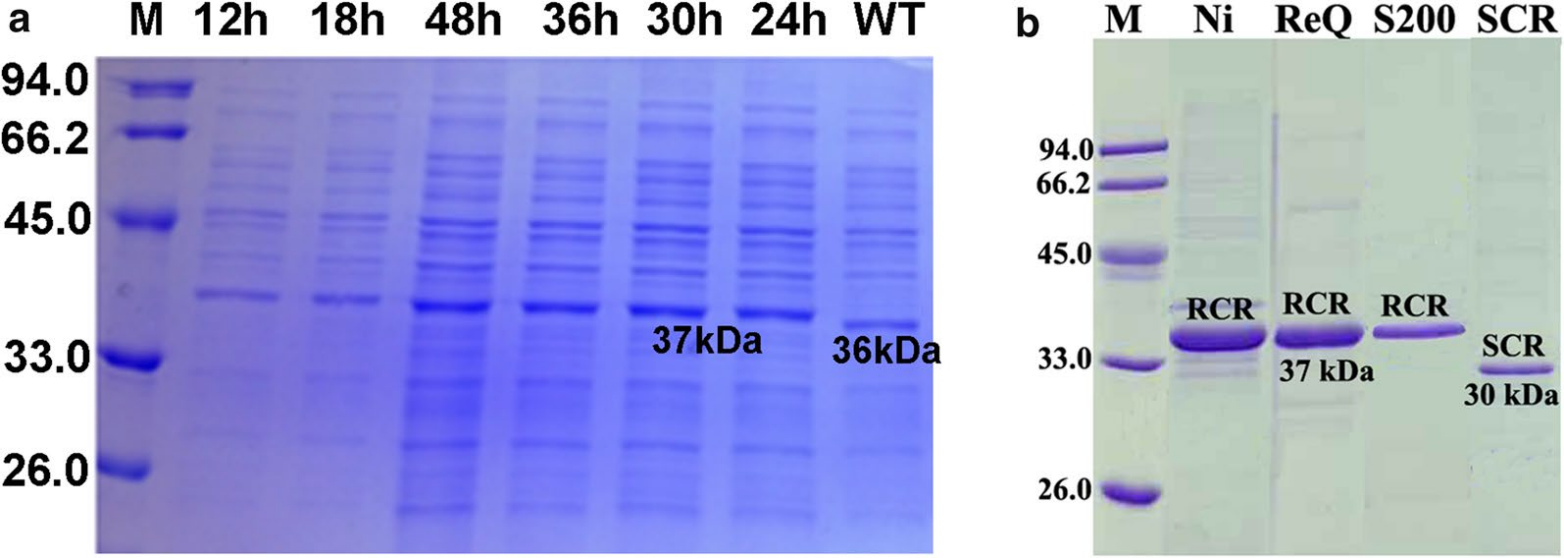
**a** SDS-PAGE analysis of RCR expression at different time points in recombinant *C*. *parapsilosis*/pCP-RCR. The cell-free extracts of *C*. *parapsilosis*/pCP-RCR collected at different time points were separated on 15 % polyacrylamide gels. *Lane M*, protein molecular standards. **b** SDS-PAGE analysis of purified RCR. *ReQ* the fraction purified by Resource Q chromatography; *S200* the fraction purified by Superdex-200 chromatography; *SCR* purified SCR from *C*. *parapsilosis*/pCP-RCR; *Lane M*, protein molecular standards

*Eco*RI linearized pCP-*rcr* plasmid was transformed into *C. parapsilosis* by electroporation. *C. parapsilosis*/pCR-*rcr* transformants were selected using nourseothricin as a positive selection marker and a uracil auxotroph as a negative selection marker. Colony PCR was carried out using RCR_1 and RCR_2 as primers, and subsequent nucleotide sequencing confirmed that the *rcr* gene was integrated into the *C. parapsilosis* genome. SDS-PAGE analysis showed that a predominant band corresponding to the expected size of the 6× His-tagged RCR enzyme (37 kDa) was observed in cell-free extracts of *C. parapsilosis*/pCR-*rcr*. The expression of recombinant RCR reached the highest level at 30 h. The expression of RCR was obviously low in WT *C. parapsilosis* with a molecular size of 36 kDa (Fig. 3a).

RCR has two functions including oxidation of (*R*)-PED to 2-HAP and reduction 2-HAP to (*R*)-PED. Recombinant *C. parapsilosis*/pCP-*rcr* showed a specific activity of 0.74 U/mg for (*R*)-PED oxidation, and 0.15 U/mg for 2-HAP reduction in the cell-free extracts, while WT *C. parapsilosis* presented 0.04 U/mg for (*R*)-PED oxidation, and 0.16 U/mg for 2-HAP reduction (Table 1). The recombinant *C. parapsilosis*/pCP-*rcr* showed 4–5 folds higher oxidative and reductive activity than WT. But the two strains presented the same activity for reduction of 2-HAP to (*S*)-PED in the cell-free extracts. The recombinant *C. parapsilosis*/pCP-*rcr* biotransformed racemic PED to its (*S*)-isomer with an optical purity of 95.6 % and a yield of 43.5 % in 30 h. Compared to WT, recombinant *C. parapsilosis*/pCP-*rcr* improved the optical purity and yield of (*S*)-PED 3.4 and 32.6 %, respectively. Notably, the biotransformation process was shortened from 48 to 30 h (Table 1).

**Table 1:** The specific activities of RCR and SCR in the cell-free extracts of recombinant *C. parapsilosis*/pCP-*rcr* and wild-type *C. parapsilosis*

Cell-free extracts	RCR^a^		SCR	Biotransformation		
	Oxidative activities (U/mg) (*R*)-PED→2-HAP	Reductive activities (U/mg) 2-HAP→(*R*)-PED	Reductive activities (U/mg) 2-HAP→(*S*)-PED	Optical purity (%)	Yield (%)	Duration (h)
*C. parapsilosis*	0.04 ± 0.00	0.16 ± 0.08	0.39 ± 0.03	92.5 ± 1.7	32.8 ± 0.8	48
*C. parapsilosis*/pCP-*rcr*	0.15 ± 0.01	0.74 ± 0.07	0.40 ± 0.04	95.6 ± 2.1	43.5 ± 1.0	30

^a^The reductive activities of RCR catalyzing (*R*)-PED to 2-HAP were determined using NADH as cofactor. The oxidative activities of RCR catalyzing 2-HAP to (*R*)-PED were determined using NAD^+^ as cofactor

RCR and SCR mediated biotransformation of racemic PED to (*S*)-PED contains two steps: RCR catalyzes (*R*)-PED to 2-HAP; SCR catalyzes 2-HAP to (*S*)-PED. The kinetic parameters of RCR catalyzing (*R*)-PED oxidation and SCR catalyzing 2-HAP reduction were assessed in WT and recombinant *C. parapsilosis*/pCP-*rcr*. The recombinant *C. parapsilosis*/pCP-*rcr* showed about two-fold higher *k*_*cat*_ value than WT, but maintained *K*_*m*_ value towards (*R*)-PED oxidation. WT and recombinant *C. parapsilosis*/pCP-*rcr* showed almost the same *K*_*m*_ and *k*_*cat*_ values towards 2-HAP reduction (Table 2). The relative *k*_*cat*_/*K*_*m*_ of RCR and SCR was 2.08 in WT *C. parapsilosis*, while it was 1.14 in *C. parapsilosis*/pCP-*rcr* (Table 2), suggesting the better rebalance of RCR and SCR functions.

**Table 2:** Kinetic parameters for oxidation of (*R*)-PED to 2-HAP by RCR and reduction of 2-HAP to (*S*)-PED by SCR in recombinant *C. parapsilosis*/pCP-*rcr* and wild-type *C. parapsilosis*

Strains	Oxidation of (*R*)-PED to 2-HAP by RCR			Reduction of 2-HAP to (*S*)-PED by SCR			Relative^a^ *k*_*cat*_/*K*_*m*_
	*K*_*m*_ (mM)	*k*_*cat*_ (S^−1^)	*k*_*cat*_/*K*_*m*_ (S^−1^ mM^−1^)	*K*_*m*_ (mM)	*k*_*cat*_ (S^−1^)	*k*_*cat*_/*K*_*m*_ (S^−1^ mM^−1^)	
*C. parasilosis*	6.53 ± 0.20	1.88 ± 0.14	0.29	4.36 ± 0.13	2.58 ± 0.05	0.59	2.03
*C. parapsilosis*/pCP-*rcr*	6.31 ± 0.09	3.19 ± 0.22	0.51	4.39 ± 0.10	2.53 ± 0.02	0.58	1.14

All reactions involved in the calculation of kinetics constants were assayed with 100 mM acetate buffer (pH 6.5) at 35 °C. All experiments were repeated three to five times

^a^Relative *k*_*cat*_/*K*_*m*_ between oxidation of (*R*)-PED to 2-HAP by RCR and reduction of 2-HAP to (*S*)-PED by SCR

### Purification of RCR and SCR and their temperature and pH dependence

Recombinant RCR with a C-terminal His_6_-tag was purified using the standard technique in the “Methods” section. The proteins were purified to apparent homogeneity by SDS-PAGE (Fig. 3b). SCR was purified from the recombinant *C. parapsilosis*/pCP-*rcr* according to methods described by Zhang et al. [17].

The pH and temperature dependences of purified RCR were determined. The recombinant RCR showed its optimal pH 5.0 and 30 °C for catalyzing (*R*)-PED to 2-HAP, and pH 6.5 and 35 °C for catalyzing 2-HAP to (*R*)-PED at pH 6.5 and 35 °C (Additional file 1: Figures S1, S2). When RCR was kept at temperatures below 40 °C for 1 h or pH 4.5–6.5 for 48 h, it retained >70 % of its maximum oxidative and reductive activity (Additional file 1: Figures S1, S2).

The enzyme SCR was purified from recombinant *C. parapsilosis*/pCP-*rcr.* It showed its optimal pH 4.5 and 35 °C for catalyzing 2-HAP to (*S*)-PED at pH 4.5 and 35 °C. When SCR was incubated at pH 4.0–7.0 for 48 h or 40 °C for 1 h, about 80 % of its maximum activity was retained (Additional file 1: Figures S3, S4).

### The biotransformation of (*R*)-PED to (*S*)-isomer under the optimum conditions of RCR and SCR

**Fig. 4:**
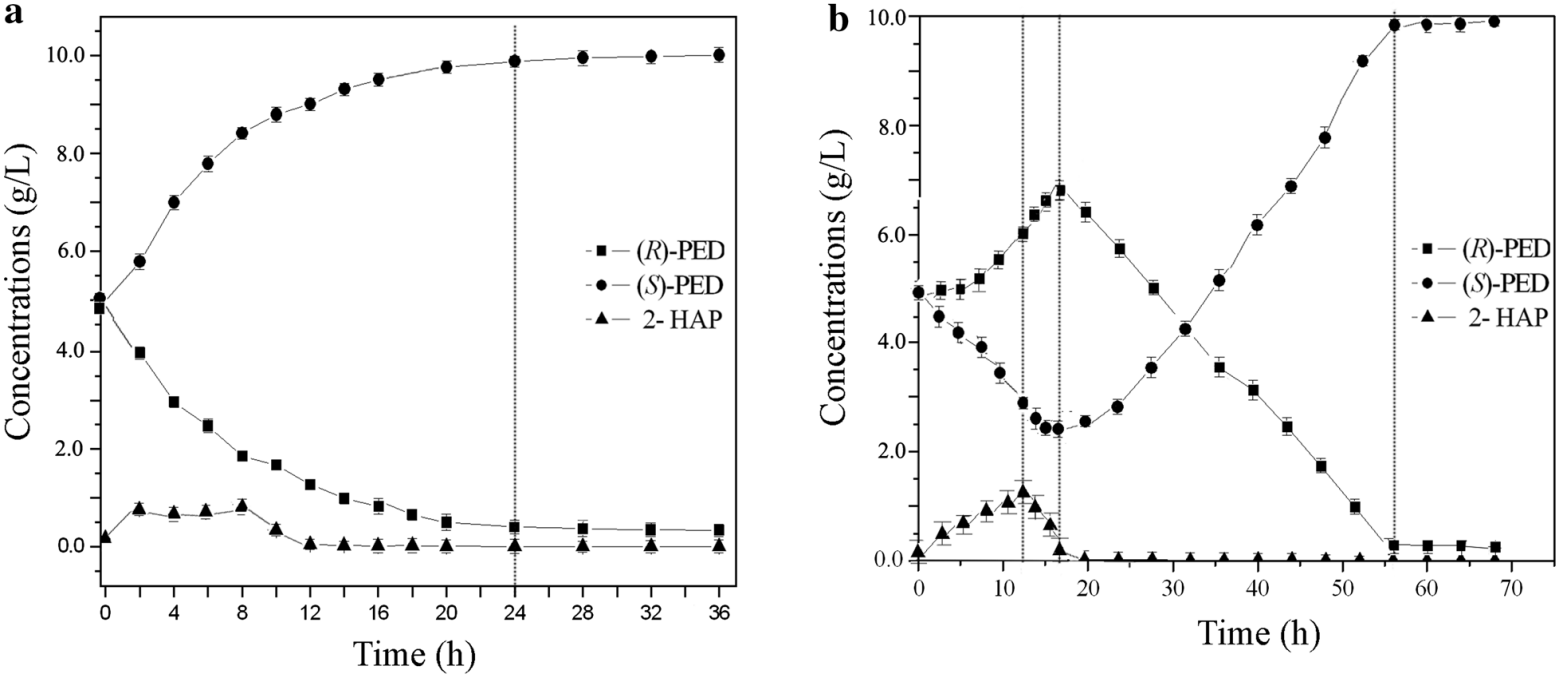
The biotransformation course of racemic (*R,S*)-PED to its (*S*)-isomer by recombinant *C*. *parapsilosis*/pCP-RCR cells. **a** Biotransformation carried out using 2-HAP as substrate; **b** biotransformation carried out using racemic (*R,S*)-PED as substrate

Under the optimum conditions for RCR catalyzing (*R*)-PED to 2-HAP (i.e., pH 5.0 and 30 °C), the recombinant *C. parapsilosis*/pCP-*rcr* cells converted racemic (*R,S*)-PED to (*S*)-PED and the reaction reached a good balance in 24 h. At that time, the consumption rate of (*R*)-PED and the formation rate of (*S*)-isomer was almost the same, and 2-HAP was maintained at a stable level (Fig. 4a). (*S*)-PED was produced with an optical purity of 93.7 % and a yield of 46.5 %.

Under the optimum conditions for RCR catalyzing 2-HAP to (*R*)-PED (i.e., pH 6.5 and 35 °C), There are three distinct stages during the biotransformation of racemic (*R,S*)-PED to (*S*)-PED by recombinant *C. parapsilosis*/pCP-*rcr*. In the initial stage (0–12 h), (*R*)-PED was transformed to 2-HAP. But the intermediate 2-HAP could not be rapidly transformed to (*S*)-PED, resulted in 2-HAP accumulation to the highest level (1.5 g/L) at 12 h (Fig. 4b). At that time, the ratio of the concentration of (*R*)-PED to (*S*)-PED was about 2:1. In the middle stage (12–16 h), the intermediate 2-HAP was almost completely consumed and transformed to (*R*)-PED. In the final stage (16–55 h), (*R*)-PED concentration gradually decreased and was transformed to 2-HAP by RCR; and 2-HAP was then rapidly converted to (*S*)-PED by SCR until the (*R*)-PED was almost completely consumed (Fig. 4b). Finally, recombinant *C. parapsilosis*/pCP-*rcr* catalyzed racemic mixture to its (*S*)-PED with an optical purity of 94.7 % and a yield of 48.1 % in 55 h.

### Significantly improved biotransformation efficiency of (*S*)-PED using a two-stage control strategy

**Fig. 5:**
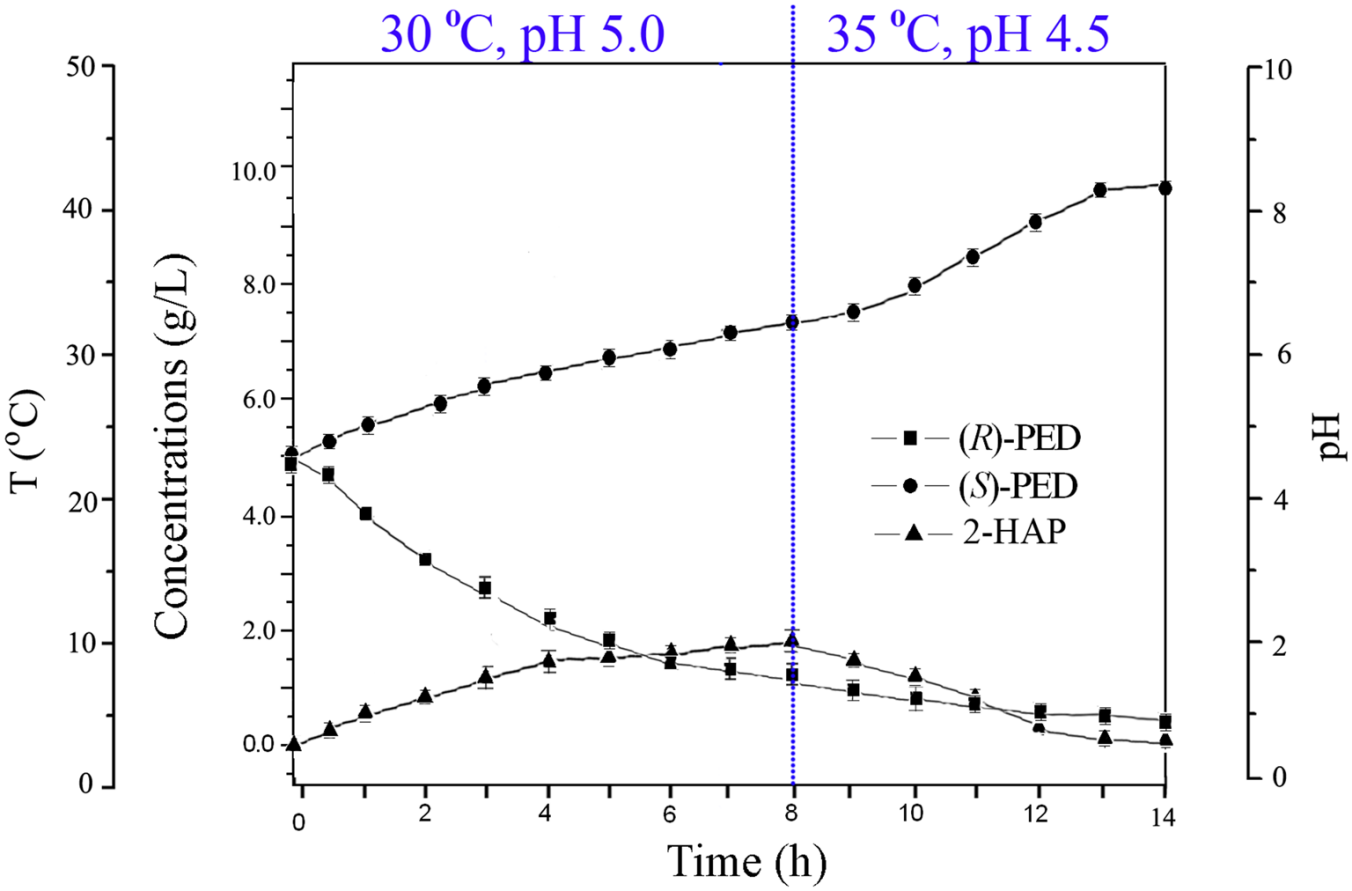
Optimized biotransformation of racemic (*R,S*)-PED to its (*S*)-isomer through a two-stage control strategy. The temperature and pH were set at 30 °C and 5.0 in 8 h, and then converted at 35 °C and 4.5 from 9 to 13 h. The recombinant *C*. *parapsilosis*/pCP-RCR cells catalyzed racemic (*R,S*)-PED to its (*S*)-isomer with a high optical purity (98.8 %) and a yield of 48.4 % within 13 h without addition of cofactors

To improve stereoinversion efficiency of racemic (*R,S*)-PED to (*S*)-PED and shorten biotransformation duration, a two-stage control strategy was devised based on the optimal conditions of two sequential reactions: RCR catalyzing (*R*)-PED to 2-HAP and SCR catalyzing 2-HAP to (*R*)-PED. In an asymmetric 10-mL reactor, the pH and temperature were initially set at 5.0 and 30 °C for RCR rapidly catalyzing (*R*)-PED to 2-HAP (Fig. 5). The initial level of 10 g/L racemic (*R,S*)-PED quickly decreased and most of substrate was consumed in 8 h by recombinant *C. parapsilosis*/pCP-*rcr*. At that time, the intermediate 2-HAP reached its highest level (1.8 g/L). Then the biotransformation conditions were adjusted to 35 °C and pH 4.5 for SCR quickly converting 2-HAP to (*S*)-PED. The residual substrate racemic (*R,S*)-PED and the intermediate 2-HAP were both consumed and transformed to (*S*)-PED from 8 h to 13 h (Fig. 5). During the final stages (from 12 to 13 h), there was few residual intermediate 2-HAP. Finally, recombinant *C. parapsilosis*/pCP-*rcr* catalyzed racemic mixture to (*S*)-PED with an optical purity of 98.8 % and a yield of 48.4 % in 13 h.

## Discussion

*Candida parapsilosis* transformes racemic PED to its (*S*)-isomer including two steps: RCR catalyzes the oxidation of (*R*)-PED to 2-HAP, and SCR catalyzes the reduction of 2-HAP to (*S*)-PED. However, *C. parapsilosis* and recombinant *E. coli*-RCR-SCR catalyzed the biotransformation of (*S*)-PED with a yield of 75–83 %, and the process required a long time (48 h) [8, 9]. By analysis of RCR and SCR functions, we found that SCR showed much higher overall activity than RCR, which may be due to its exhibiting more correct protein folding and higher protein expression in *C. parapsilosis* and other host cells [8, 29]. Moreover, a cluster of four SCR isozymes was recently found in *C. parapsilosis* [30], which enlarged the activity gap between RCR and SCR.

In this work, we designed a special expression plasmid pCP by cloning the expression components into pUC57, and introduced an in situ expression system of RCR into *C. parapsilosis* to strengthen RCR function. To improve screening probability of the positive *C*. *parapsilosis*/pCP-*rcr* clones, the RCR expression cassette was designed using nourseothricin as a positive selection marker and a uracil auxotroph as a negative selection marker. The minimal inhibitory concentration of nourseothricin was 60 μg/ml in *C. parapsilosis*. Reuss et al. used the drug nourseothricin as a dominant selectable marker for gene disruption in *C. albicans* [31]. Shen et al. used the nourseothricin adapted to suit *C. albicans* in a Ca-NAT1–FLP cassette [32]. However, the concentration of nourseothricin used in *C. albicans* was different from that of in *C. parapsilosis* which may be due to the different resistance levels of two *Candida* strains. Using this screening technique, we selected the clones with the highest activities from about 120 positive *C. parapsilosis* clones.

The constitutive expression of 6× his tagged RCR strengthened its function in *C. parapsilosis*. The recombinant *C. parapsilosis*/pCP-*rcr* showed over fourfold higher activities for catalyzing (*R*)-PED to 2-HAP than WT, which may be due to the integration of several copies of the RCR gene into the *C. parapsilosis* genome [21, 33]. The introduction of in situ expression of RCR had no effect on the cell-growth and SCR activity. The in situ expression of RCR did not change *K*_*m*_ value towards (*R*)-PED oxidation, and *K*_*m*_ and *k*_*cat*_ values towards 2-HAP reduction, but increased *k*_*cat*_ value towards (*R*)-PED oxidation about two times. The relative *k*_*cat*_/*K*_*m*_ of SCR catalyzing 2-HAP and RCR catalyzing (*R*)-PED was about 2 in WT *C. parapsilosis*, indicating the imbalance between RCR and SCR functions, with SCR performing at a higher rate than RCR. The relative *k*_*cat*_/*K*_*m*_ value was about 1 in *C. parapsilosis*/pCP-*rcr*, suggesting good rebalance between RCR and SCR functions. The obviously higher *k*_*cat*_/*K*_*m*_ value of RCR in *C. parapsilosis*/pCP-*rcr* than that of in WT may be due to the 6× histine tag at N-terminal of RCR in *C. parapsilosis*/pCP-*rcr*. Yeon et al. thought that the catalytic activity of 3-hydroxybutyrate dehydrogenase was not severely affected by the N-terminal His-tag due to the location of the N-terminus far from the active site of the enzyme [34]. Dickson et al. reported that the N-terminus His-tag did result in a differential effect on protein kinase activity, further potentiating the elevated protein kinase activity of both the helical domain and catalytic domain oncogenic mutants with relation to p110 phosphorylation [35]. The introduction of in situ expression of RCR better rebalanced RCR and SCR functions, resulting in obviously improved optical purity and yield of (*S*)-PED during the biotransformation of racemic PED to its (*S*)-isomer. Compared to WT, the recombinant *C. parapsilosis*/pCP-*rcr* reduced the biotransformation duration from 48 to 36 h.

To further improve the stereoconversion efficiency of racemic (*R,S*)-PED to its (*S*)-isomer by recombinant *C. parapsilosis*/pCP-*rcr*, the biotransformation process should be optimized. Since RCR not only catalyzes oxidation of (*R*)-PED to 2-HAP, but also catalyzes reduction of 2-HAP to (*R*)-PED. The latter function is unfavorable for the biotransformation of racemic PED to (*S*)-PED. By analysis of the whole biotransformation process, we found much time was wasted in the reversible reaction between 2-HAP and (*R*)-PED. Basing on pH and temperature preferences of RCR catalyzing (*R*)-PED to 2-HAP and SCR catalyzing 2-HAP to (*S*)-PED, we envisaged a two-stage control strategy to optimize the asymmetric biotransformation process. The biotransformation was first carried out under the optimal conditions for RCR rapidly catalyzing (R)-PED to 2-HAP, then under the optimal conditions for SCR converting 2-HAP to (S)-PED. Using these strategies, the recombinant *C. parapsilosis*/pCP-*rcr* strains efficiently converted racemic (*R,S*)-PED to the (*S*)-isomer with good performance: an optical purity of 98.8 % and a yield of 48.4 %. Since racemic (*R,S*)-PED contains 50 % (*S*)-PED, the final yield of (*S*)-PED by the recombinant *C. parapsilosis*/pCP-*rcr* is 98.4 %. Zhang et al. reported one-step biotransformation of (*R*)-PED to (*S*)-isomer by an enzyme-coupled system *E. coli*/pET-RCR-SCR. The system produced (*S*)-PED with an optical purity of 91.3 % and a yield of 75.9 % in 48 h [8]. Nie et al. enhanced the biotransformation efficiency of (*R,S*)-PED to its (*S*)-isomer with an optical purity of 98.2 % and a yield of 82.9 % in 48 h using agitation speed control strategy during cell cultivation of *C. parapsilosis* CCTCC 203011 [9]. Compared with the *E. coli*/pET-RCR-SCR and WT *C. parapsilosis*, the recombinant *C. parapsilosis*/pCP-*rcr* increased the yield of (*S*)-PED about 18.7–29.3 %. More importantly, the biotransformation time was reduced about four-fold: from 48 to 13 h [8, 9].

## Conclusions

There is much imbalance between the activities of RCR and SCR, resulting in a low biotransformation efficiency of racemic (*R,S*)-PED to its (*S*)-isomer by *C. parapsilosis*. In this work, we designed an in situ expression plasmid, pCP, and expressed RCR in *C. parapsilosis* to narrow the function gap between RCR and SCR. Then we developed a two-stage control strategy based on pH and temperature preferences of RCR and SCR. The in situ expression system biotransformed racemic (*R,S*)-PED to its (*S*)-isomer with good performance: an optical purity of 98.8 % and a yield of 48.4 %. Most notably, the biotransformation duration was reduced about four times: from 48 to 13 h with respect to the WT. This work provides a method for improving chiral biosynthesis efficiency through in situ expression of a rate-limiting enzyme and a two-stage control strategy to rebalance asymmetric pathways. Further research will be carried out using the safer microbial hosts for more efficient protein expression and chiral biosynthesis.

## Methods

### Chemicals

NADPH and 2-HAP were obtained from Sigma-Aldrich Chemical Co., Inc. (St. Louis, USA). Restriction enzymes, nourseothricin, uracil and 5-fluoroorotic acid were obtained from Takara Bio. Co. (Kyoto, Japan). All other chemicals were of the high grade commercially obtainable.

### Organisms and culture conditions

*Candida parapsilosis* CCTCC M203011 was obtained from the American Type Culture Collection. It was cultured in yeast extract peptone-dextrose medium (1 % yeast extract, 2 % glucose, 2 % peptone) at 30 °C. *E. coli* DH5α was grown in lysogeny broth medium (0.5 % yeast extract, 1 % NaCl, 1 % peptone) at 37 °C. When nesseary, 60 μg/ml nourseothricin and 20 μg/ml uracil was added. Recombinant *C. parapsilosis* were cultured in medium containing 0.5 % yeast extract, 4 % maltose, 1.3 % (NH_4_)_2_HPO_4_, 0.7 % KH_2_PO_4_, 0.01 % NaCl, and 0.08 % MgSO_4_·7H_2_O with the addition of 60 μg/ml nourseothricin and 20 μg/ml uracil.

### Construction of the in situ expression plasmid pCP-*rcr*

Plasmids, strains and primers used in this work are listed in Additional file 1: Table S1 in the supplemental material. The *SAT1*-flipper method and *ura3*-negative method were used to construct the RCR in situ expression plasmid for *C. parapsilosis*. The promoter *act*1A gene (GenBank ID: AJ389062.1) was amplified from the *C. parapsilosis* genome using primers ACT1p_1 and ACT1p_2, and was cloned into the corresponding *Not*I*/Hin*dIII sites of pMD19-T (Takara Co., Dalian, China), resulting in the recombinant plasmid T-*act1p*. The *sat1* gene (KF318043.1) (of 525-bp) was amplified with primers SAT1_1 and SAT1_2 (*Not*I*/Bam*HI). The *ura3* (3636649) downstream fragment (508 bp) was cloned using primers URA3t_1 and URA3t_2 (*Bam*HI/*Hin*dIII). The *sat1*-*ura3t* fragment was generated using a modified overlap-extension technique [36] with the primers SAT1_1 and URA3t_2. The *Not*I*/Hin*dIII fragment of *sat1*-*ura3t* was inserted into plasmid T-*act1p* to generate the plasmid T-*act1p*-*sat1*-*ura3t*. The upstream sequence of *ura3* (285-bp) was amplified with primers URA3p_1 and URA3p_2 (*Eco*RI*/Bgl*II), and the *mal2* promoter (3638946) (548 bp) was amplified using primers MAL2p_1 and MAL2p_2 (*Bgl*II*/Sac*I). The *ura3p*-*mal2p* fragment was generated using primers URA3p_1 and MAL2p_2. The *Eco*RI/*Sac*I fragment of *ura3p*-*mal2p* was inserted into pUC57 to generate plasmid pUC57-*ura3p*-*mal2p*. The *rcr* gene (GenBank ID: DQ295067) with a 6× His-tag at the C-terminus was amplified from the *C. parapsilosis* genome with primers RCR_1 and RCR_2 (containing recognition sites for *Sac*I and *Kpn*I), and cloned into pUC57-*ura3p*-*mal2p* to generate pUC57-*ura3p*-*mal2p*-*rcr*. The *act1* downstream sequence (388-bp) was amplified with primers ACT1t_1 and ACT1t_2 (containing recognition sites for *Kpn*I and *Pst*I), and cloned into pUC57-*ura3p*-*mal2p*-*rcr*, generating pUC57-*ura3p*-*mal2p*-*rcr*-*act1t*. The plasmid T-*act1p*-*sat1*-*ura3t* was digested with *Pst*I and *Hin*dIII and then inserted into pUC57-*ura3p*-*mal2p*-*rcr*-*act1t*, generating the recombinant plasmid pUC57-*ura3p*-*mal2p*-*rcr*-*act1t*-*act1p*-*salt*-*ura3t*, named pCP-*rcr*. All the recombinant plasmids were verified by DNA sequencing.

### Electrotransformation of *C. parapsilosis*

*Candida parapsilosis* cells were cultured overnight until the *OD*_600_ value reached 2.0, and were then inoculated into 50 ml yeast extract peptone-dextrose medium at 30 °C. When the *OD*_600_ value of the culture reached 1.0–2.0, the culture was resuspended in 50 ml of Tris–EDTA buffer (pH 7.5) containing 10 mM dithiothreitol, and was then kept on ice for 30 min. After the cells were washed four times using 10 ml of 1 M ice-cold sorbitol, the competent cells were resuspended in 80 μl 1 M sorbitol. The vector pCP-*rcr* was linearized with *Eco*RI and then transformed into *C. parapsilosis* with a MicroPulser electroporator (Bio-Rad, USA) at 2000 V in a 1-mm cuvette. After electroporation, 1 ml of ice-cold 1 M sorbitol was immediately added. The cells were incubated at 30 °C for 2 h and collected at 4000×*g* for 10 min. After resuspension in 100 μl sorbitol (1 M), the cultures were plated onto minimal dextrose plates containing 60 μg/ml nourseothricin and 20 μg/ml uracil. The *C. parapsilosis*/pCR-*rcr* transformants were obtained after incubation for 48 h.

### Cell-free extract preparation

Recombinant *C. parapsilosis* cells were grown for 48 h in medium containing 0.5 % yeast extract, 4 % maltose, 1.3 % (NH_4_)_2_HPO_4_, 0.7 % KH_2_PO_4_, 0.01 % NaCl, and 0.08 % MgSO_4_·7H_2_O. When necessary, 60 μg/ml nourseothricin was added into the medium. The cells were harvested by centrifugation and washed twice with 0.8 % NaCl solution, and then suspended in 0.1 M potassium phosphate buffer (pH 6.5). WT *C. parapsilosis* and recombinant *C. parapsilosis*/pCR-*rcr* cells were harvested and broken using an APV 2000 homogenizer (SPX Co. Unna, Germany). The homogenate was centrifuged at 48,000×*g* for 40 min. The supernatant of WT *C. parapsilosis* and recombinant *C. parapsilosis*/pCR-*rcr* was used for further enzyme assay and protein purification.

### Enzyme assay

The mixture for assaying reductive activity of RCR contained 0.1 M potassium phosphate buffer (pH 6.5), 0.5 mM NADH/NADPH, 5 mM 2-HAP, and an appropriate amount of RCR or the cell-free extracts of WT and recombinant *C. parapsilosis*/pCR-*rcr* in a total volume of 100 µl. The mixture for assaying oxidative activity of RCR comprised 0.1 M potassium phosphate buffer (pH 6.5), 0.5 mM NAD^+^, 5 mM (*R*)-PED, and an appropriate amount of RCR or cell-free extracts of WT and *C. parapsilosis*/pCR-*rcr* in a total volume of 100 µl. The decrease/increase in the concentration of NAD(P)^+^ or NAD(P)H was recorded spectrophotometrically at 340 nm. The reduction activity of SCR was measured as described by Zhang et al. [13]. One unit of enzyme activity was defined as the amount of enzyme catalyzing the oxidation/reduction of 1 µmol NAD(P)H/NAD(P)^+^ per minute in the assay conditions. The protein concentration was determined using Bradford reagent (Bio-Rad) with bovine serum albumin as the standard [37]. Reported values represent the average of at least three independent measurements.

### Temperature and pH dependence of enzymes

The temperature dependence of enzyme activity was determined from 10 to 60 °C. The pH dependence of the reductive activity was determined between pH 4.0 and 9.0 using 100 mM citrate buffer (pH 3.0–4.5), 100 mM sodium acetate buffer (pH 4.0–6.0), 100 mM potassium phosphate buffer (pH 6.0–7.0) or 100 mM Tris–HCl buffer (pH 7.0–9.0) at 35 °C. The enzyme activity was otherwise measured with the standard assay method described above.

### Stability of enzymes

Thermostability was determined by incubation of the purified RCR and SCR (0.2 mg/ml) in 100 mM NaAC-HAC buffer, pH 6.0, at temperatures between 10 and 60 °C. The pH stability of enzymes was determined by incubating purified enzyme at pH 3.0–9.0 and 35 °C. After exposure of enzyme to the indicated conditions for 1 h, samples were taken to assay for residual activity.

### Protein purification

Recombinant RCR were expressed in *C. parapsilosis* with a His_6_-tag. Cell-free fractions were applied to Ni^2+^-Sepharose affinity chromatography columns (HisTrap Kit, GE Healthcare, Uppsala, Sweden) using an ÄKTA Protein Purifier system. The pooled fractions were then loaded onto a Resource Q column (1 × 1 cm) equilibrated with 20 mM Tris–HCl (pH 8.0). Finally, the fractions were applied to a Superdex 200 column (HiLoad 26/60, preparation grade) for chromatography in a buffer containing 20 mM Tris–HCl (pH 8.0) and 150 mM NaCl. The homogeneity of purified enzymes was judged by Coomassie Brilliant Blue staining of SDS-PAGE.

### Determination of kinetic parameters

To determine the kinetic parameters, various concentrations of substrate 2-HAP or (*R*)-PED (0.5–20 mM), enzyme (10–200 μM), and cofactors NAD^+^ or NADPH (0.5–5.0 mM) in 0.1 M NaAC-HAC buffer (pH 6.5) at 35 °C were used. The data were fitted to the Michaelis–Menten equation by using a nonlinear least-square iterative method using KaleidaGraph (Synergy Software, Reading, PA). Three sets of kinetic parameters were obtained from three independent experiments and then simply averaged to yield the final estimates. The final estimates are shown with the standard errors for the three sets.

### Biotransformation and analytical methods

The biotransformation was carried out as previously described [9] with minor modifications. The reaction mixture in 1 ml contained 0.1 M potassium phosphate buffer (pH 6.5), 10 mg racemic (*R,S*)-PED, and 0.1 g wet cells at 35 °C, or 0.1 M NaAC-HAC buffer (pH 5.0), 10 mg racemic (*R,S*)-PED, and 0.1 g wet cells at 30 °C. Reactions were carried out for 48 h with shaking at 220 rpm. Through a two-stage pH and temperature control strategy, the reaction mixture in 10 ml contained 0.1 M potassium phosphate buffer, 10 mg racemic (*R,S*)-PED, and 1.0 g wet cells. The biotransformation was carried out at pH 5.0 and 30 °C for 8 h, then at pH 4.5 and 35 °C for 5 h. The products were extracted with ethyl acetate and the organic layer was analyzed by high-performance liquid chromatography on a Chiralcel OB-H column (Daicel Chemical Ind. Ltd., Japan). The conditions for the HPLC analyses—mobile phase, hexane:isopropanol 9:1; flow rate: 0.4 ml/min, operating column temperature: 38 °C; and UV detector. The optical purity and yield of products were calculated using the following equations [9]:

$$ \begin{aligned} {\text{Optical purity}}_{{(R){\text{-PED}}}} \\ &= {{\left( {(R){\text{-PED\,-\,}}(S){\text{-PED}}} \right)} / {\left( {(R){\text{-PED}}\text\,{+}\,(S){\text{-PED}}} \right)}}  \\&\quad \times 100\,{\% } \end{aligned} $$

$$ {\text{Yield}}_{{(R){\text{-PED}}}} = ( R) {\text{-PED/2-HAP}} \times 1 0 0\,{\% } $$

(*R*)-PED, (*S*)-PED and 2-HAP are measured in grams.

## Additional file

10.1186/s12934-016-0539-y Supplemental data containing plasmids, list of primers and strains and additional results.

## Abbreviations

2-HAP: 2-hydroxyacetophenone
PED: 1-phenyl-1,2-ethanediol
RCR: (*R*)-carbonyl reductase
SCR: (*S*)-carbonyl reductase
