# Supplementary material for: In situ expression of (R)-carbonyl reductase rebalancing an asymmetric pathway improves stereoconversion efficiency of racemic mixture to (S)-phenyl-1,2-ethanediol in Candida parapsilosis CCTCC M203011
Source: Microb Cell Fact. 2016 Aug 17;15:143. doi: 10.1186/s12934-016-0539-y (PMC4989518; doi:10.1186/s12934-016-0539-y)
Supplement: Supplementary file 1 — 10.1186/s12934-016-0539-y Supplemental data containing plasmids, list of primers and strains and additional results. [file 12934_2016_539_MOESM1_ESM.doc]

## *In* *situ* expression of (*R*)-carbonyl reductase rebalancing an asymmetric pathway improves stereoconversion efficiency of racemic mixture to (*S*)-phenyl-1,2-ethanediol in *Candida parapsilosis* CCTCC M203011

Rongzhen Zhang^1,2^*, Lei Wang^1^, Yan Xu^1,2^*, Hongbo Liang^1^, Xiaotian Zhou^1^, Jiawei Jiang^1^, Yaohui Li^1^, Rong Xiao^3^

**Table S1. Strains, plasmids and primers used in this work**

| **Plasmids, strains and primers** | **Description** | **Sources** |
| --- | --- | --- |
| **Strains** |  |  |
| *E*. *coli* DH5α (DE3) | Host cell for gene cloning | Invitrogen |
| *C. parapsilosis* CCTCC M203011 | Source of *rcr* gene and host cell for *in-situ* expression of *rcr* | This work |
| *E. coli* DH5α/pCP | *E. coli* DH5α harboring pCP | This work |
| *E. coli* DH5α/pCP-*rcr* | *E. coli* DH5α harboring pCP-*rcr* | This work |
| *C. parapsilosis*/pCP-*rcr* | *C. parapsilosis* harboring pCP-*rcr* | This work |
|  |  |  |
| **Plasmids** |  |  |
| pMD19-T | The cloning plasmid of *rcr* | Invitrogen |
| pUC57 | Source of *in-situ* expression plasmid pCP | This lab |
| pCP | The *in-situ* expression in *C. parapsilosis* | This work |
| T-*rcr* | pMD19-T harboring *rcr* | This work |
| pCP-*rcr* | pCP harboring *rcr* | This work |
|  |  |  |
| **Primers** | (5’ → 3’) |  |
| URA3p_1 | ccggaattcgtattgcaaacaaacg | This work |
| URA3p_2 | cgcatccattcagatcttgtatgaagacggca | This work |
| MAL2p_1 | gtcttcatacaagatctgaatggatgcggg | This work |
| MAL2p_2 | attgacatgagctcccgcggaatagttgtagta | This work |
| RCR_1 | ctacaactattccgcgggagctcatgtcaattccatca | This work |
| RCR_2 | cactcggtaccctagtggtggtggtggtggtgtggattaaaa | This work |
| ACT1t_1 | caccactagggtaccgagtgaaattct | This work |
| ACT1t_2 | gtcttcctgcagattttatgatggaat | This work |
| ACT1p_1 | aaaatctgcaggaagaccgtccaac | This work |
| ACT1p_2 | tttaagcttatctgcggccgcaccgttatcgataactaaa | This work |
| SAT1_1 | cgataacggtgcggccgcatgaaaatttcggtga | This work |
| SAT1_2 | gattaaatattcggatccttaggcgtcatcct | This work |
| URA3t_1 | gatgacgcctaaggatccgaatatttaatcat | This work |
| URA3t_2 | atcccaagcttaacgatcaagagaaa | This work |

**Notes:** The underlined sequences are the restriction sites.

**Figure S1. Effect of pH on activity and stability of the recombinant RCR**

**A, B**: Effects of pH on RCR for 2-HAP reduction and (*R*)-PED oxidation. **C, D**: Stability of RCR for 2-HAP reduction and (*R*)-PED oxidation.

The recombinant RCR activity was measured using standard assay procedure in the following 0.2 M buffers containing: (i) citrate buffer (pH 3.0-4.5); (ii) acetic acid buffer (pH 4.5-6.0); (ii) phosphate buffer (pH 5.5-8.0); (iii) Tris–HCl buffer (pH 8.0-9.0). Relative activity was expressed as a percentage of maximum activity under experimental conditions.

**
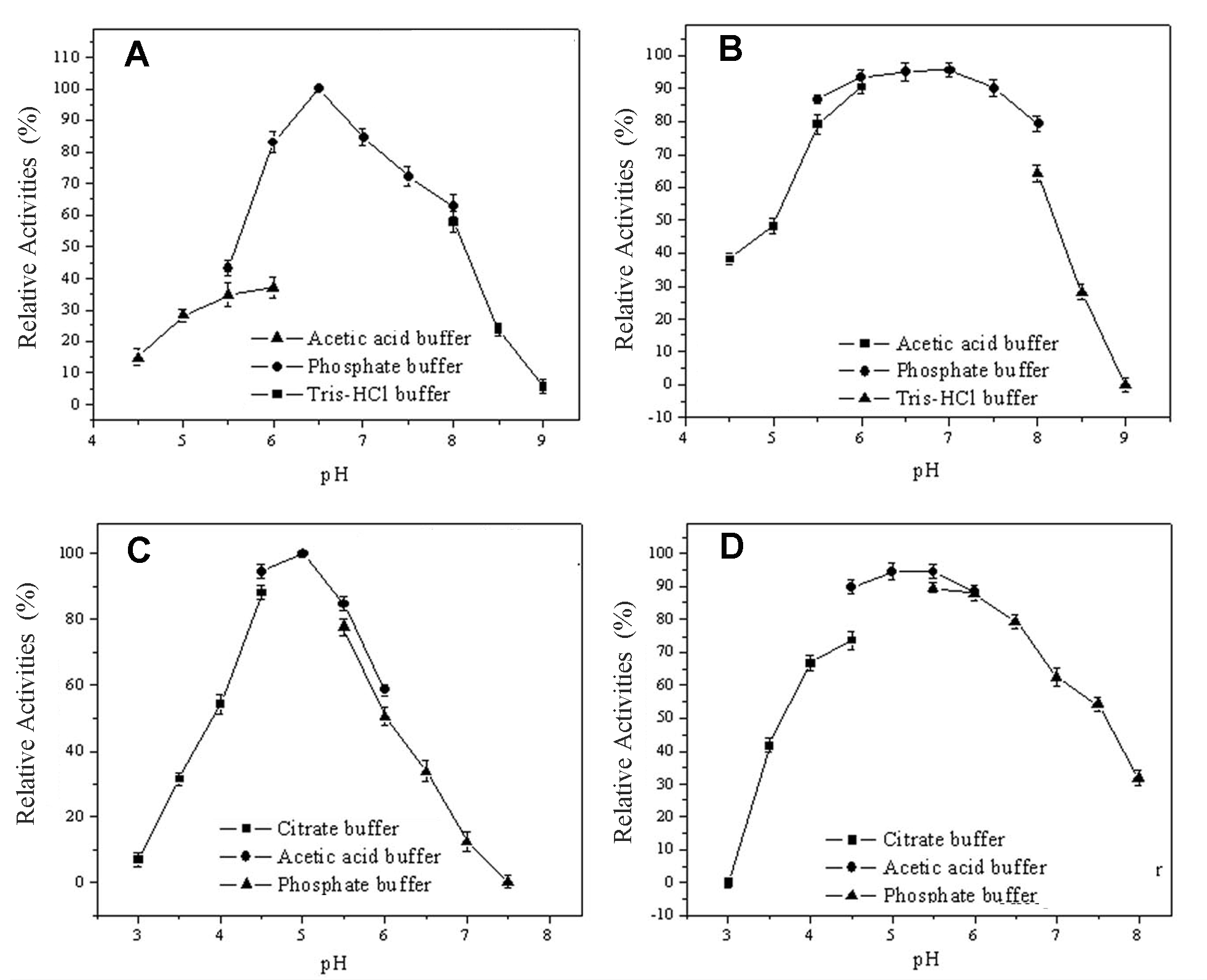
**

**Figure S2. Effect of temperature on activity and stability of recombinant RCR**

Enzyme assay was performed using standard assay procedure at various temperatures between 10-60℃ in 0.2 mol/L phosphate buffer (pH 6.5 for reduction and pH 5.0 for oxidation) for 1 h. The relative activity was expressed as percentage of maximum activity under experimental condition. **A, B**: Effects of temperature on RCR activity for 2-HAP reduction and (*R*)-PED oxidation. **C, D**: Stability of RCR for 2-HAP reduction and (*R*)-PED oxidation at different temperature.

**
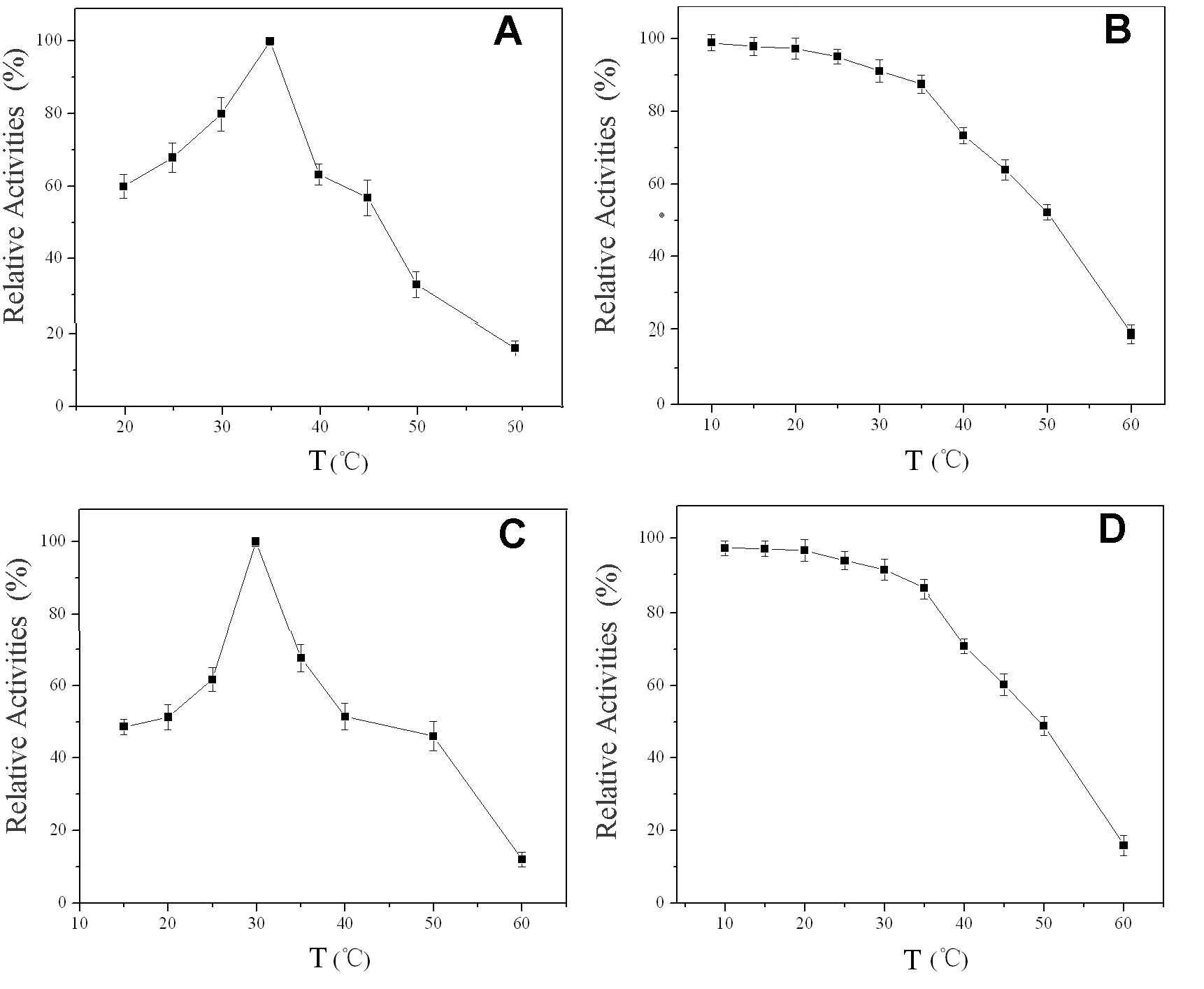
**

**Figure S3. Effect of pH on activity and stability of SCR**

**A**: Effects of pH on SCR for 2-HAP reduction. **B**: Stability of SCR for 2-HAP reduction at different pH values.

The activity of recombinant SCR was assayed using standard assay procedure in the following 0.2 mol/L buffers containing: (i) citrate buffer (pH 3.0-4.5); (ii) acetic acid buffer (pH 4.5-6.0); (ii) phosphate buffer (pH 5.5-8.0); (iii) Tris–HCl buffer (pH 8.0-9.0). Relative activity was expressed as a percentage of maximum activity under experimental conditions.

**
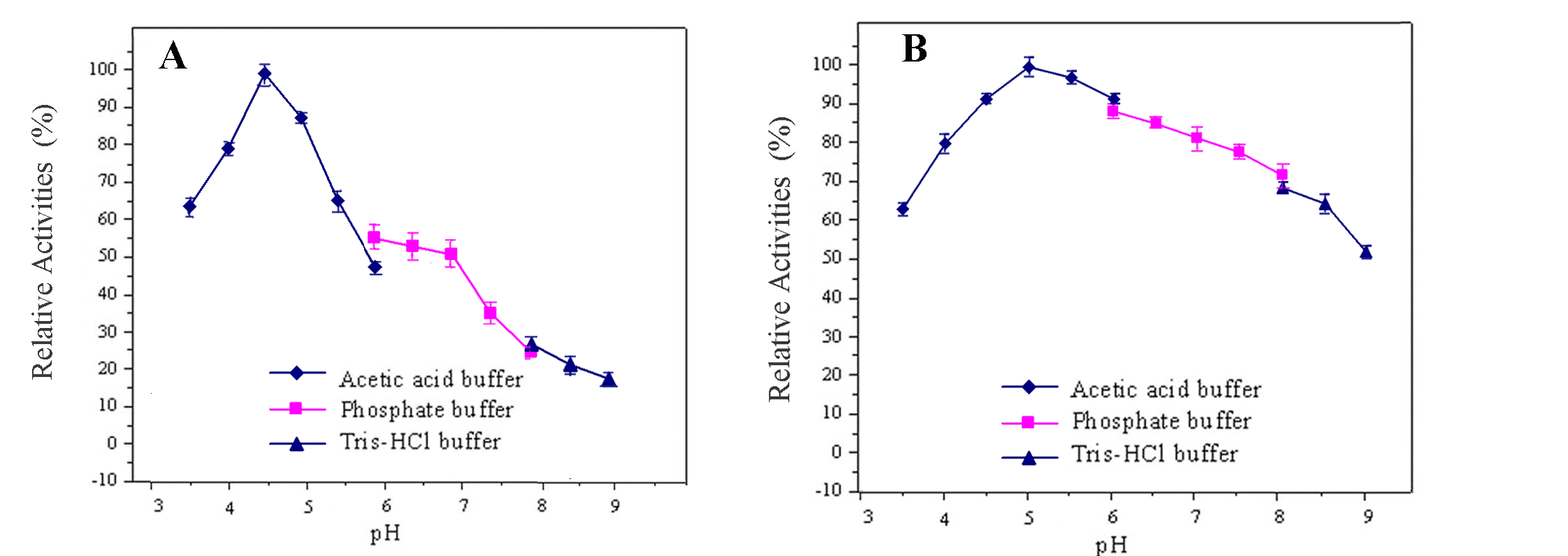
**

**Figure S4. Effect of temperature on activity and stability of SCR**

Enzyme assay was performed using standard assay procedure at various temperatures between 10-70℃ in 0.2 mol/L phosphate buffer (pH 6.5) for 1 h. The relative activity was expressed as percentage of maximum activity under experimental condition. **A**: Effects of temperature on SCR for 2-HAP reduction. **B**: Stability of SCR for 2-HAP reduction at different temperatures.

**
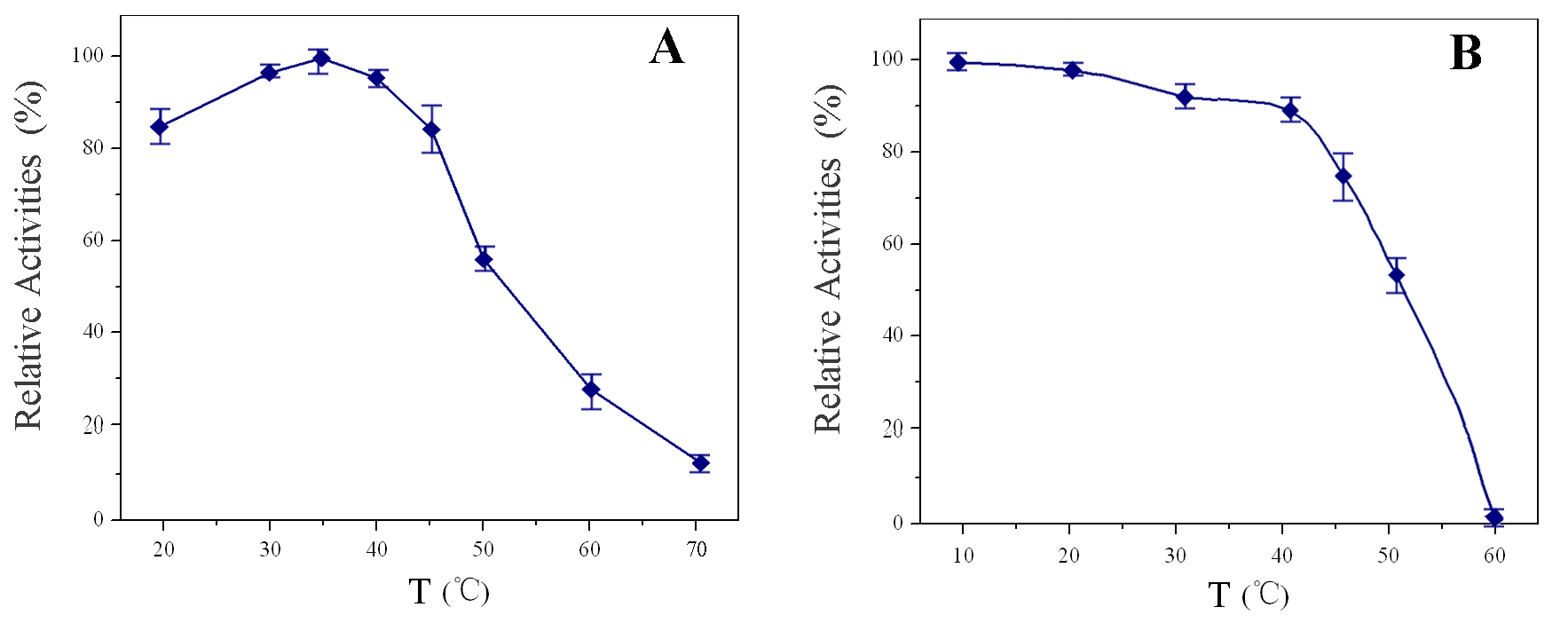
**
